# Chronic MK-801 Application in Adolescence and Early Adulthood: A Spatial Working Memory Deficit in Adult Long-Evans Rats But No Changes in the Hippocampal NMDA Receptor Subunits

**DOI:** 10.3389/fphar.2018.00042

**Published:** 2018-02-12

**Authors:** Libor Uttl, Tomas Petrasek, Hilal Sengul, Marketa Svojanovska, Veronika Lobellova, Karel Vales, Dominika Radostova, Grygoriy Tsenov, Hana Kubova, Anna Mikulecka, Jan Svoboda, Ales Stuchlik

**Affiliations:** ^1^Department of Developmental Epileptology, Institute of Physiology, Czech Academy of Sciences, Prague, Czechia; ^2^Department of Experimental Neurobiology, National Institute of Mental Health, Klecany, Czechia; ^3^Department of Neurophysiology of Memory, Institute of Physiology, Czech Academy of Sciences, Prague, Czechia; ^4^Radboud Institute for Molecular Life Sciences, Radboud University, Nijmegen, Netherlands; ^5^Second Faculty of Medicine, Charles University, Prague, Czechia

**Keywords:** schizophrenia, animal model, dizocilpine, rats, chronic treatment, western blot, behavior

## Abstract

The role of NMDA receptors in learning, memory and hippocampal function has long been recognized. Post-mortem studies have indicated that the expression or subunit composition of the NMDA glutamate receptor subtype might be related to the impaired cognitive functions found in schizophrenia patients. NMDA receptor antagonists have been used to develop animal models of this disorder. There is accumulating evidence showing that not only the acute but also the chronic application of NMDA receptor antagonists may induce schizophrenia-like alterations in behavior and brain functions. However, limited evidence is available regarding the consequences of NMDA receptor blockage during periods of adolescence and early adulthood. This study tested the hypothesis that a 2-week treatment of male Long-Evans and Wistar rats with dizocilpine (MK-801; 0.5 mg/kg daily) starting at postnatal days (PD) 30 and 60 would cause a long-term cognitive deficit and changes in the levels of NMDA receptor subunits. The working memory version of the Morris water maze (MWM) and active place avoidance with reversal on a rotating arena (Carousel) requiring cognitive coordination and flexibility probed cognitive functions and an elevated-plus maze (EPM) was used to measure anxiety-like behavior. The western blot method was used to determine changes in NMDA receptor subunit levels in the hippocampus. Our results showed no significant changes in behaviors in Wistar rats. Slightly elevated anxiety-like behavior was observed in the EPM in Long-Evans rats with the onset of treatment on PD 30. Furthermore, Long-Evans rats treated from PD 60 displayed impaired working memory in the MWM. There were; however, no significant changes in the levels of NMDA receptor subunits because of MK-801 administration. These findings suggest that a 2-week treatment starting on PD 60 in Long-Evans rats leads to long-term changes in working memory, but this deficit is not paralleled by changes in NMDA receptor subunits. These results support the face validity, but not construct validity of this model. We suggest that chronic treatment of adolescent and adult rats does not constitute a plausible animal model of schizophrenia.

## Introduction

Schizophrenia is a devastating neuropsychiatric disease (Owen et al., 2016), affecting all populations worldwide with a lifetime prevalence of approximately 1%. Beside, to its well-known symptoms, this disorder manifests with severe cognitive deficits. Importantly, although cognitive deficits used to be viewed as secondary symptoms, today they are considered the most stable symptom class and provide the most rigorous basis for predictions of long-term treatment outcomes (Elvevag and Goldberg, 2000; Owen et al., 2016). It has long been suspected that the glutamatergic system of the brain is involved in schizophrenia (Kantrowitz and Javitt, 2012) because many glutamatergic markers are altered in the brains of schizophrenic patients. More recently, a decreased function of glutamate receptors in schizophrenia has been combined with developmental concepts involving changes in genetic and environmental contributing to the disease to form a neurodevelopmental hypothesis of schizophrenia (Davis et al., 2016).

As no direct causes or causal treatments for schizophrenia are known, scientists often develop and evaluate animal models of schizophrenia (Jones et al., 2011) as tools for investigating mechanisms that may play a role in the actual disease and to search for novel drugs with a better risk/benefit ratio. Importantly, marked disruptions of behavioral functions similar to those found in schizophrenia can be induced in animal models using acute application of MK-801, a prototypical experimental high-affinity non-competitive antagonist of NMDA receptors. These include social deficits (low doses; Rung et al., 2005), cognitive deficits (low-to-moderate doses; van der Staay et al., 2011; Lobellova et al., 2013, Kubík et al., 2014; Svoboda et al., 2015) and toxic and experimental psychoses (higher doses, Vales et al., 2006; Lobellová et al., 2015). Chronic experiments aimed at mimicking the neurodevelopmental abnormalities have shown that these manipulations can also induce schizophrenia-like behaviors. There is a large body of evidence in rats treated with non-competitive NMDA receptor antagonists at an early postnatal age (excellently reviewed by Lim et al., 2012). However, relatively fewer studies have focused on the chronic effects of NMDA receptor antagonism in subsequent ontogenetic periods such as late adolescence or early adulthood, despite the fact that schizophrenia in human patients manifests itself most commonly in this age range (but see Li et al., 2011).

We hypothesized that repeated administration of MK-801 adolescent (starting on PD 30) and young adult rats (PD 60) would result in cognitive disturbances and changes in the levels of NMDA receptor subunits in the hippocampus, a region crucial for these functions and critically involved in schizophrenia. We sought to remedy knowledge gap on this time of treatment and test phenomenological (changes in behavior) and construct (changes in NMDA receptor subunits) axes of validity of this model.

## Materials and Methods

### Animals

We use two common outbred rat strains, Wistar and Long-Evans. Male rats of Wistar (*n* = 32) and Long-Evans (*n* = 40) strains were obtained from the breeding colony of the Institute of Physiology of the Czech Academy of Sciences (IPHYS). They were housed in 25 cm × 30 cm × 40 cm plastic transparent cages in groups consisting of 2–4 animals in an air-conditioned animal room with constant temperature, humidity and a 12/12 h light/dark cycle. The rats were weaned at PD 28. Access to food and water was always *ad libitum*. All animal manipulations were approved by the Ministry of Agriculture committee and done according to the approved project of experiments no. 136/2013. The procedures complied with the Animal Protection Code of Czechia and the appropriate directive of the European Union (2010/63/EC).

### Drugs

MK-801 (dizocilpine maleate) was obtained from Sigma–Aldrich, Czechia. The drug was dissolved in saline at a concentration of 0.5 mg/ml and injected subcutaneously in the skin fold between the shoulders at a dose of 0.5 mg/kg body weight. Control groups received subcutaneous injections of saline at a volume of 1 ml/kg. Injections were administered between 10 am and 11 am for 14 consecutive days.

### Design of Study

For behavioral testing, we employed a working memory version of the MWM (Vales et al., 2006) with 15-s intervals between swims. Active place avoidance with reversal on a rotating arena (Carousel) was used to test cognitive coordination and flexibility (for review see Stuchlík et al., 2013). For assessment of anxiety-like behavior, we used an EPM. Notably, working memory, cognitive coordination and behavioral flexibility are markedly disrupted in patients with schizophrenia (Elvevag and Goldberg, 2000; Owen et al., 2016). Moreover, we analyzed levels of protein for NMDA receptor subunits in the hippocampus. We detected the GluN1, GluN2A, and GluN2B subunits by western blot analyses to assess changes in their expression.

Animals from each strain were injected with MK-801 starting at two ages: PD 30 or PD 60. Age-matched controls were given saline. All Long-Evans groups consisted of 10 animals. All Wistar groups consisted of eight animals. Animals were assigned to treatment groups randomly prior to start of experiments. Long-Evans and Wistar rats were tested in two different runs, separated by a 2-month interval; therefore, no direct comparisons of behavioral parameters between those two strains were performed.

**FIGURE 1:**
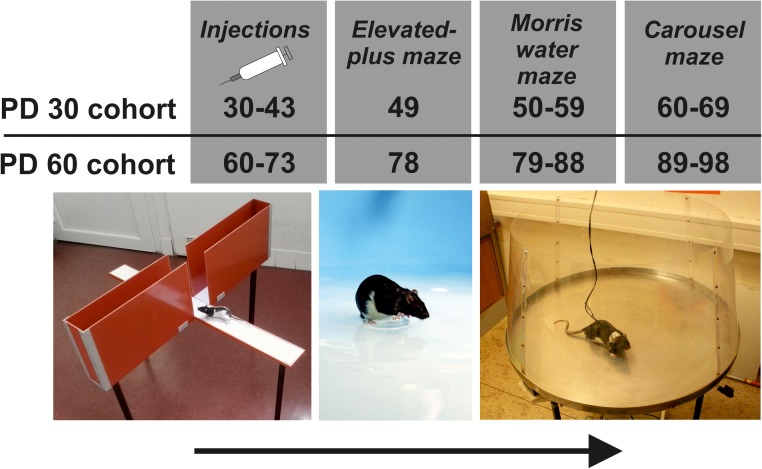
Schematic diagram of the experimental design.

Injections were separated by 24 h. Upon completing the injections, animals were left undisturbed for 5 days in their home cages to stabilize their behavior and for the drug to wash out, to prevent acute side effects from affecting the results. The behavioral tests were performed in the following sequence: the EPM, the working memory version of the MWM, and active place avoidance with reversal (**Figure 1**). At the age of 3 months, animals were decapitated in isoflurane anesthesia and both right and left hippocampi were dissected and prepared for western blot analysis of the levels of NMDA receptor subunits (see the section Bioanalytical Analysis below).

### Behavioral Tasks

#### Elevated Plus Maze

The EPM is a gold standard for testing anxiety-like behavior in rodents (Haller et al., 2013). Many patients with schizophrenia report increased anxiety possibly do to the presence of positive symptoms (Temmingh and Stein, 2015), that is why we used this test as a part of behavioral battery aiming at face validity of chronic treatment with MK-801.

The EPM was a test of unconditioned avoidance behavior that involved four narrow arms elevated one meter above the floor of a dimly lit experimental room. A standard elevated plus maze apparatus was used (Pellow et al., 1985). Two opposite arms were enclosed by walls and the remaining arms were open. In the standard 5-min version of the test, rats spent more time exploring the enclosed arms than the open. Each rat was placed in the center of the maze with the head pointing toward the closed arms, and was then allowed to move freely for 5 min. Viewer (Biobserve) and Ethovision (Noldus) software was used to quantify behavior. The EPM test was administered on PD 49 in animals with the onset of treatment on PD 30, and on PD 78 in animals treated from PD 60 (**Figure 1**).

#### Working Memory Version of the Morris Water Maze

The working memory version of the MWM (Vales et al., 2006) permits the study of spatial working memory encoded by a single learning trial (the first swim in each session). Working memory deficit constitutes a strong and reliable phenotype for schizophrenia (Forbes et al., 2009) and reaches spatial domain (Fajnerová et al., 2014). We therefore used this task as a component of battery assessing face validity of chronic MK-801 model. The MWM was performed over 10 daily sessions consisting of four swims (trials) to a hidden platform, with a delay of 15 s between trials. The platform position was changed daily in a pseudorandom order. Each day, the platform was in a unique location; there was to interference between the sessions. Release positions were pseudo-randomized by the partial Latin square method. The MWM was administered from PD 50 in the younger age cohort and from PD 79 in the older age cohort (**Figure 1**).

#### Active Place Avoidance on a Rotating Arena (Carousel)

Active place avoidance with reversal in Carousel is a test of cognitive coordination and behavioral flexibility. The task has been repeatedly validated (Bures et al., 1997; Fenton et al., 1998; Czéh et al., 2001; Stuchlik and Bures, 2002; Wesierska et al., 2005; Kubík et al., 2006; Stuchlik and Vales, 2006; Petrasek et al., 2014a,b; for review see Stuchlík et al., 2013) and a typical dynamic-environment test (Stuchlik, 2014; for comparison, see Telensky et al., 2011). To our best knowledge, no other test places demands for coordinating two reference frames (arena and room frame). Cognitive coordination is significantly disrupted in schizophrenia (Phillips and Silverstein, 2003); therefore, the selection of this task was justified in this study. The testing consisted of 10 sessions (all separated by 24-h intervals). The initial five sessions were considered as acquisition sessions (with the to-be-avoided sector in arbitrary North), which were followed by five reversal sessions, with the sector position shifted by 180 degrees (arbitrary South). This shift made the task sensitive not only to cognitive coordination, but also to cognitive flexibility (Burghardt et al., 2012; Lobellova et al., 2013; Svoboda et al., 2015). Carousel maze testing was conducted during daylight hours (10 am – 4 pm).

The rotating arena (Carousel) (invented by Fenton et al., 1998 and originally described by Bures et al., 1997) was a smooth metallic arena (82 cm in diameter), enclosed with a 50-cm transparent Plexiglas wall (for details of the apparatus and procedures see Stuchlík et al., 2013). Prior to testing, conscious rats were gently implanted with a hypodermic needle, piercing the skin fold on the animal’s back. The sharp end of needle was blunted and a small loop was created with tweezers, preventing the needle from slipping out and providing purchase for an alligator clip, which delivered mild electric shocks (see below). The needle implantation corresponded to subcutaneous injection in humans and did not require anesthesia. At the beginning of each session, a rat was placed on the arena, which rotated constantly at one revolution per minute. A 60-degree to-be-avoided sector was defined in the coordinate frame of the room by a computer-based tracking system (Tracker, Biosignal Group, United States), which also recorded the positions of the rat and the arena (which were both marked by infrared LED diodes) at a sampling rate of 25 Hz. Each entrance into the sector lasting more than 300 ms was punished by mild constant-current footshocks (repeated every 1200 ms until the rat left the sector) delivered by the tracking system. The intensity of the shock was individualized for each rat (0.2–0.6 mA), to ensure an escape reaction while avoiding freezing. Most rats responded appropriately to 0.2 or 0.4 mA. The footshocks were administered through a cable attached to a harness on the back of the rat and connected to the conductive subcutaneous implant. This level of shock intensity is set to be unpleasant for rats, but not painful; in the latter case, the footshock application would elicit freezing, which would have prevented the avoidance learning. Therefore, we kept the footshock on minimal possible level. The trajectories were digitized and recorded on a PC, allowing the off-line reconstruction and analysis of the animal’s trajectory and avoidance behavior with Track Analysis software (Biosignal Group, United States). Further detailed analysis and verification of the data was done in the open-source software Carousel Maze Manager (Bahník, 2014). Testing in the Carousel was conducted from PD 60 in the younger age cohort and from PND 89 in the older age cohort (**Figure 1**).

### Bioanalytical Analysis

Western blot analysis was performed to determine changes in the concentration of subunits of the NMDA receptor, measuring the levels of protein expression for the GluN1, GluN2A, and GluN2B subunits. Tissue from the left and right hippocampi was collected from both the Wistar and Long-Evans rats, frozen, and stored at -80°C until analysis. Next, all samples were homogenized using a series of ultrasonic pulses (50% of max. amplitude, duration 500 ms, 8 pulses per sample; UP100H, Hielscher) with 10 mM PBS (pH 7.4) at a ratio of 1:9 and protease inhibitor cocktail (#P8340, Sigma–Aldrich). Obtained homogenates were centrifuged (#120951, SIGMA 2-16 PK) at 1000 *g* for 10 min at 4°C and the supernatant was collected. A small volume of the hippocampus lysate was used for the quantification of protein concentrations by the Lowry method (Lowry et al., 1951) with Peterson’s modification (Peterson, 1977).

Before electrophoresis, samples were mixed with Laemmli loading buffer (#161-0737, Bio-Rad, Hercules, CA, United States) and heated for 20 min at 70°C. Stain-Free gradient gels (#567-8084, Bio-Rad, Hercules, CA, United States) were used for protein separation. The samples were subsequently transferred to nitrocellulose membranes (#170-4271, Bio-Rad) using a *Trans*-blot Turbo apparatus (Bio-Rad, United States). The quality of transfer and volume of protein on the membrane were determined by a ChemiDoc^TM^ Touch Imaging System (Bio-Rad, United States). Membranes were blocked in 5% non-fat milk in TBS overnight. On the subsequent day, membranes were first incubated with either the primary antibodies anti-GluN1 1:1000 (NeuroMab clone N308/48), anti-GluN2A 1:500 (NeuroMab clone N327/95) or anti-GluN2B 1:1000 (NeuroMab clone N59/36), and then washed 3x 10 min in TBS. Then, membranes were incubated for 2 h at room temperature with secondary antibody at 1: 30 000 (#115-035-174, Jackson ImmunoResearch Laboratories, Baltimore, PA, United States) for 1 h (RT) and washed with TBS as described above. A chemiluminescent substrate (Supersignal West Femto, #34096, Thermo Scientific, Waltham, MA, United States) was used for the visualization of protein with the ChemiDoc^TM^ Touch Imaging System (Bio-Rad, United States). The bands were detected and analyzed with ImageLab software. (Bio-Rad, United States). Stain-free images of total protein were used for normalization of the target proteins as described previously (Tramutola et al., 2016). When performing normalization, detection of a housekeeping protein (for example, actin, tubulin, GAPDH) or total protein in the lines can be used. However, housekeeping proteins can vary in different tissues, at different ages, but also because of experimental manipulations (Fukazawa et al., 2003; Li and Carmichael, 2006). For these reasons, we chose normalization to total protein. The above-mentioned stain-free method was used to determine the total protein in the lanes. The principle of the method is based on modification of the tryptophan amino acid by the compound trihalo, resulting in a very marked increase in fluorescence after activation by UV light. This method is comparable to protein staining with SYPRO Ruby, Coomassie Blue or silver stain (Gilda and Gomes, 2013). For illustration, see the stain-free images in Supplementary Material.

### Data Analysis and Statistics

#### Analysis of the Behavioral Parameters

In the elevated plus-maze, the following parameters were measured: time spent in the open arms, closed arms, and central platform, head dipping and risk assessments (the latter two are not reported, as no effects were seen). In the MWM, latency and total distance to reach the platform were recorded. In the Carousel, we measured the number of entrances into the to-be-avoided sector (number of errors) and total distance walked during a session (computed as the cumulative total distance of data points measured at 1-s intervals; this sampling eliminates non-locomotor movements such as shivering). We also recorded the maximum time between two entrances (maximum time avoided) and the latency to the first entrance to the to-be-avoided sector in a given session (latency to the first error). However, since these parameters generally correlate highly with the number of errors, their analysis did not provide any additional information value; these data are not included here. In all these behavioral experiments, statistical analyses were performed separately for each strain as both strains were testing in separate batches. Data from the elevated plus-maze were assessed using two-way ANOVA with age and MK-801 treatment as main factors. To avoid three-way ANOVAs in water maze and active place avoidance in which repeated measures add another factor of complexity, we selected a parameter that well characterizes the extent of learning. In water maze, we summed the latency and total distance from swims 2 to 4 and averaged them across the 10 days of training. In active place avoidance, we summed number of entrances and total distance across the 5 days of acquisition. We also evaluated these two parameters in 1st day of reversal when they best reflect behavioral flexibility. Two-way ANOVA was conducted with treatment (MK-801, saline) and age (PD30, PD60) as the main factors. When appropriate, ANOVA was followed by Sidak’s *post hoc* test. All calculations were performed using GraphPad Prism 7.0, with a level of acceptance set at *P* < 0.05. Data are reported in the figures as means ± SEM.

#### Analysis of Biochemical Parameters

Images of target proteins and stain-free images of total protein were created on the ChemiDoc^TM^ Touch system and analyzed in ImageLab. The values were taken from the left and right hippocampi, but since there were no differences between them, they were pooled together for subsequent analyses. The measured values were tested for outliers by calculating a *z* score, and consequently the values having a *z* score greater than 2 were excluded. We used 14–16 samples in each group. For statistical analysis we used Statistica 9 (StatSoft) and graphs were created using GraphPad Prism 5.0. Differences between groups were evaluated by a two-way ANOVA with treatment and age as independent factors; statistically significant differences are reported at *P* < 0.05. Data are reported as means ± SEM total protein normalized intensity.

## Results

### Repeated Administration of MK-801 Causes Severe Qualitative Acute and Long-term Effects

In both strains, both age groups of rats administered with 0.5 mg/kg MK-801 displayed acute behavioral changes as expected. Since 0.5 mg/kg is a relatively high dose, we observed the onset of restlessness 5 min after application followed by hyperactivity (running near the wall, sniffing in the corners, salivation) and motor disturbances (inability to stand upright, falling and crawling). Some rats were hypoactive and even had signs of ataxia. This type of behavior typically diminished 1–2 h after MK-801 administration. One week after injection, three Long-Evans rats exhibited increased aggressiveness, and therefore were placed in cages separately and were not included in behavioral and bioanalytical testing.

### Behavioral Results

#### Repeated MK-801 Led to Mild Long-term Anxiety-Like Behavior in Adolescent Long-Evans Rats

##### Wistar rats

**FIGURE 2:**
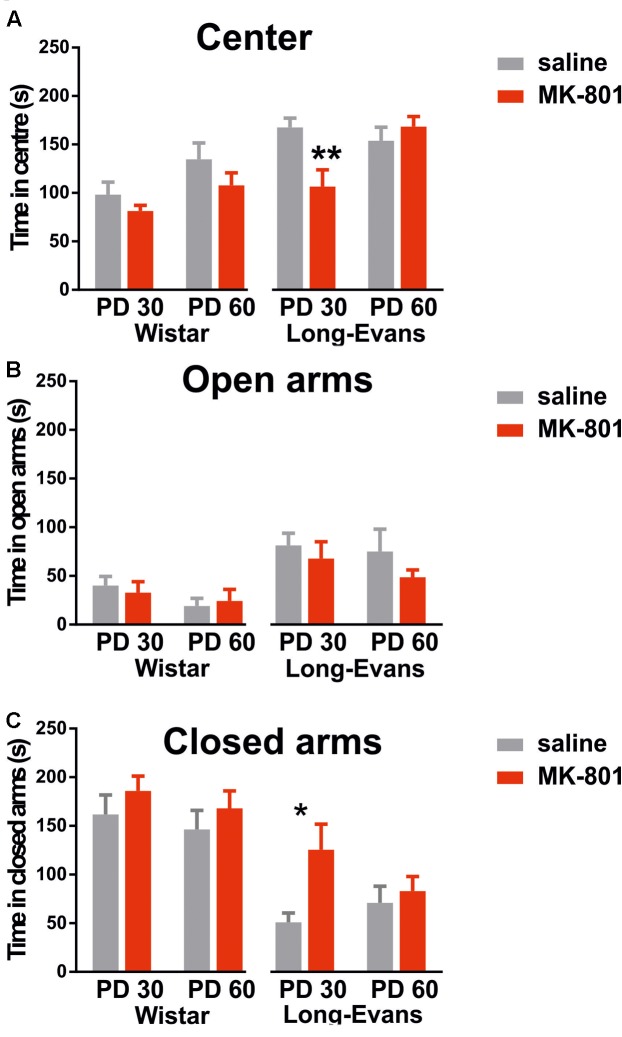
Performance in the elevated plus maze evaluated by total time spent in the central part **(A)**, open arms **(B)**, and closed arms **(C)**. Comparing MK-801 and saline-treated rats in each cohort yielded significant differences only for PD 30 Long-Evans rats in entering the center, and for PD 30 Long-Evans rats in entering the closed arms. Group means ± SEM (^∗^*P* < 0.05, ^∗∗^*P* < 0.01).

Data obtained in the EPM suggests that MK-801 exerted no effects on anxiety-like behavior in Wistar rats. As **Figure 2** illustrates, there was no difference between saline and MK-801 injected groups in neither age when evaluating the time spent in the center [*F*(1,28) = 2.988], open arms [*F*(1,28) = 0.01] or closed arms [*F*(1,28) = 1.577]. However, on average PD30 rats spent less time in the center than PD60 rats [effect of age: *F*(1,28) = 6.125, *P* = 0.0196].

##### Long-Evans rats

When analyzing behavior of Long-Evans rats, we found significant effect of MK-801 in the time spent in closed arms [*F*(1,36) = 5.737, *P* = 0.0219] and significant age vs. treatment interaction in time spent in the center [*F*(1,36) = 8.259, *P* = 0.0068]. *Post hoc* tests revealed it was due to different effects in PD30 groups. These results indicate elevated anxiety-like behavior in Long-Evans rats in the younger age cohort. On the other hand, time spent in open arms was not affected by MK-801 treatment in Long-Evans rats [*F*(1,36) = 1.556, *P* = 0.2204].

#### Repeated MK-801 Induced Long-term Impairments of Working Memory in Adult Long-Evans Rats

##### Wistar rats

**FIGURE 3:**
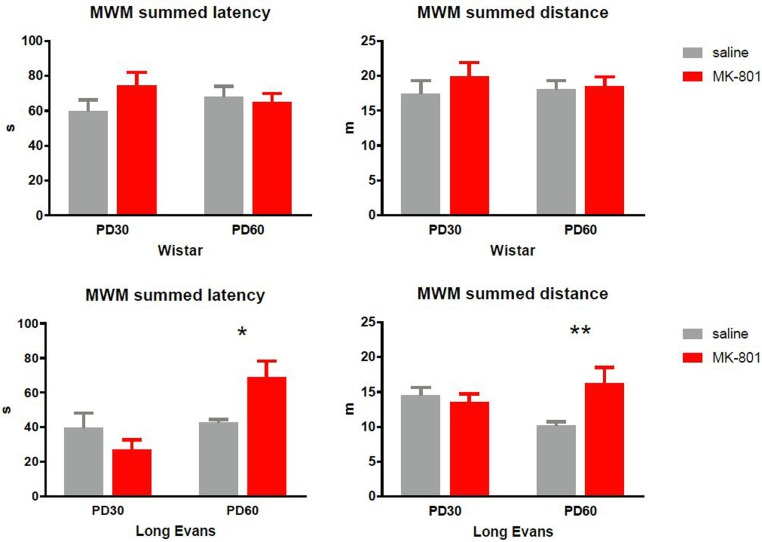
Latency and path to the platform (distance) in the water maze summed for swim 2, 3, and 4, and averaged across the 10 days of training. In the Long-Evans PD 60 cohort, MK-801 treated rats did not search for the platform on repeated swims as efficiently as saline treated rats (^∗^*P* < 0.05, ^∗∗^*P* < 0.01). Group means ± SEM.

To quantify performance in the working memory version of the MWM, we summed the latency and total distance from swims 2 to 4 and averaged them across the 10 days of training. As can be seen in **Figure 3**, MK-801 elicited no effects in Wistar rats. Two-way ANOVA failed to find an effect of MK-801 [latency: *F*(1,28) = 0.8502, *P* = 0.36; distance: *F*(1,28) = 0.8165, *P* = 0.37] and age [latency: *F*(1,28) = 0.008, *P* = 0.93; distance: *F*(1,28) = 0.056, *P* = 0.82].

##### Long-Evans rats

In contrast, pattern in Long-Evans rats was more complex. Analyzing latency, two-way ANOVA failed to see an effect of MK [*F*(1,30) = 0.96, *P* = 0.33] but showed main effect of age [*F*(1,30) = 10.52, *P* = 0.003] and interactions [*F*(1,30) = 7.99, *P* = 0.008]. Then Sidak’s *post hoc* test confirmed significantly increased latency in MK-801-treated PD 60 rats compared to PD 60 controls. Similar results were obtained when analyzing path: Main effect of MK-801 [*F*(1,33) = 4.13, *P* = 0.05], age [*F*(1,33) = 0.39, *P* = 0.54], and interaction [*F*(1,33) = 7.71, *P* = 0.009]. Subsequent *post hoc* test confirmed elevated path in PD 60 MK-801 rats compared to PD60 controls.

#### Repeated MK-801 Failed to Induce Deficits in Acquisition and Reversal Learning in the Carousel

Number of errors (entrances) reflects the ability of properly locating the to-be-avoided place on the arena. We summed number of entrances across the five sessions of acquisition to get an overall parameter of acquisition. Then we analyzed data from first reversal session as this time point allows for evaluating the ability of cognitive flexibility.

##### Wistar rats

**FIGURE 4:**
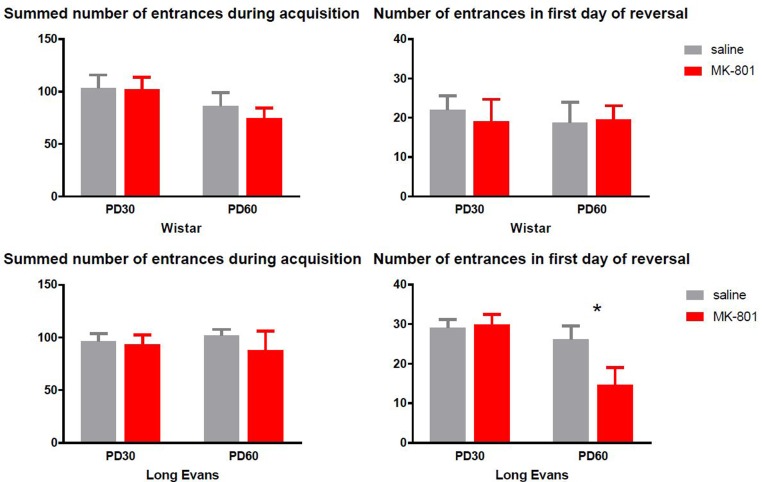
Performance in active place avoidance evaluated as the summed number of entrances into the to-be-avoided sector during 4 days of acquisition (left graphs) and 1st day of reversal (right graphs). Differences between MK-801 and saline treated rats were statistically significant only during reversal in the Long-Evans PD 60 cohort, with MK-801 administered rats to be superior to saline controls (^∗^*P* < 0.05). Group means ± SEM.

Despite **Figure 4** indicates that PD 60 Wistar rats accumulated lower number of entrances during acquisition, two-way ANOVA failed to see an effect of age [*F*(1,28) = 3.534, *P* = 0.07]. Furthermore, there was neither effect of MK-801 administration [*F*(1,28) = 0.3092, *P* = 0.58] nor significant treatment vs. age interaction – [*F*(1,28) = 0.175, *P* = 0.6789]. Similarly, cognitive flexibility as measured by number of entrances in the 1st day of reversal was not affected by MK-801 administration in Wistar rats [*F*(1,33) = 0.013, *P* = 0.91] or age [*F*(1,28) = 3.534, *P* = 0.0706]; interaction [*F*(1,28) = 0.175, *P* = 0.6789].

##### Long-Evans rats

As **Figure 4** illustrates, Long-Evans rats performed equally during acquisition at both ages [*F*(1,33) = 2.723, *P* = 0.11] despite MK-801 administration [*F*(1,33) = 0.01319, *P* = 0.9093] measured by a summed number of entrances during acquisition. Surprisingly, a two-way ANOVA found a significant effect of age [*F*(1,33) = 8.782, *P* = 0.0056] and effect of age vs. MK-801 interaction [*F*(1,33) = 3.973, *P* = 0.05] in the 1st day of reversal resulting from decreased number of entrances in Long-Evans rats treated with MK-801 from PD 60 (Sidak’s *post hoc* test, *P* = 0.03). Besides the number of entrances, we also evaluated the total distance moved during a session to investigate the effects of repeated administration of MK-801 on overall locomotion, but two-way ANOVA did not reveal a significant effect of MK-801 or age in any experimental condition in either strain or any cohort.

### Repeated MK-801 Failed to Affect the Levels of NMDA Receptors Subunits in the Hippocampus

**FIGURE 5:**
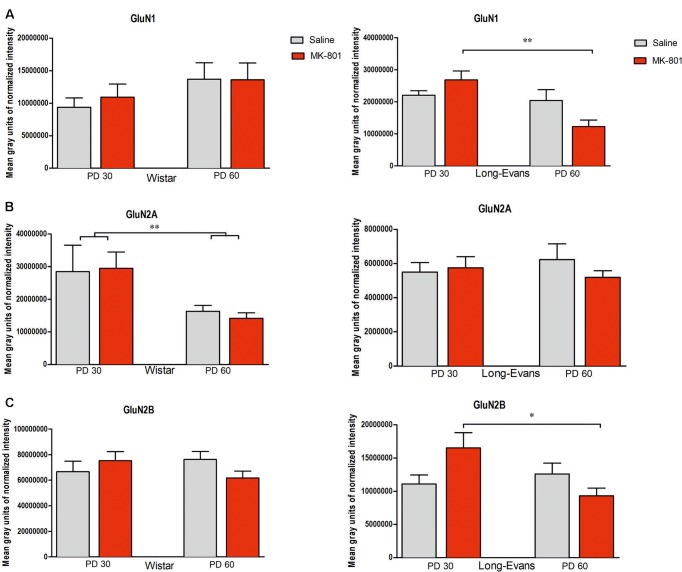
Expression of NMDA subunits in the hippocampus: **(A)** GluN1 subunit, **(B)** GluN2A subunit **(C)** GluN2B subunit: The charts show the two intervals of the start of administration (PD 30 and PD 60). Strains of rats are marked as Long-Evans (LE) and Wistar (W). The data show the mean level of chemiluminescence after total protein normalization (group means ± SEM). ^#^*P* < 0.05 compared to controls of the same strain and age. ^∗^*P* < 0.05, ^∗∗^*P* < 0.01; comparison between age cohorts.

**FIGURE 6:**
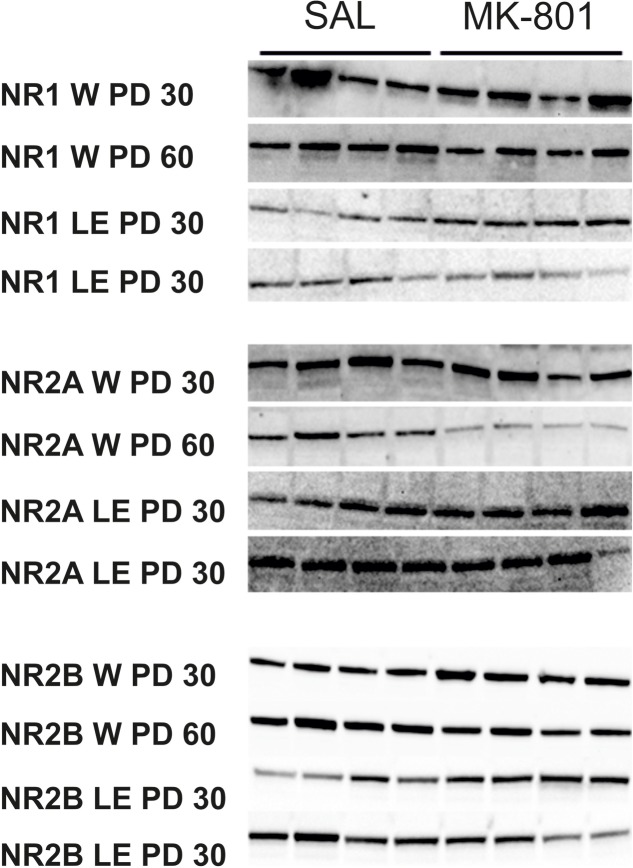
Examples of Western blot of the hippocampal supernatant showing expression of the NMDA subunits (GluN1 ∼100 kDa, GluN2A∼170 kDa, GluN2B ∼180 kDa) at two age intervals (PD 30, PD 60) in Long-Evans and Wistar rats. Control group samples are in columns 1–4 and MK-801 samples are in the columns 5–8.

**Table 1** shows the mean of values of protein concentrations (μg/μl). Hippocampal expression of NMDA subunits (GluN1, GlunN2A, and GluN2B) was analyzed for both strains independently with using a two-way ANOVA (see **Figures 5**, **6**).

**Table 1:** Mean of total protein concentration in samples for Western blot analyses.

Groups		Concentration of proteins in samples (μg/μl)		Count of samples
			
		Mean	SEM	
Wistar	Control PD 30	7.56	0.31	*n* = 16
	Control PD 60	8.02	0.13	*n* = 16
	MK-801 PD 30	7.57	0.35	*n* = 16
	MK-801 PD 60	9.18	0.25	*n* = 16
Long-Evans	Control PD 30	7.48	0.30	*n* = 20
	Control PD 60	6.23	0.34	*n* = 20
	MK-801 PD 30	6.90	0.36	*n* = 18
	MK-801 PD 60	5.42	0.49	*n* = 10


*Data was obtained by using a modificated Lowry’s method.*

#### Wistar Rats

Analysis of expression of NMDA receptor subunits in Wistar rats showed no significant changes. Expression of GluN1 showed no significant main effects of treatment group [*F*(1,60) = 0.113, *P* = 0.738] and age [*F*(1,60) = 2.556, *P* = 0.11723] neither age × treatment of interaction [*F*(1,60) = 0.141, *P* = 0.709]. Expression of GluN2A showed no significant changes main effects of age [*F*(1,60) = 2.5006, *P* = 0.11906] and treatment [*F*(1,60) = 0.8581, *P* = 0.358] neither age × treatment interaction [*F*(1,60) = 0.2149, *P* = 0.6447]. Expression of GluN2B showed no significant main effects, treatment [*F*(1,60) = 0.185, *P* = 0.668] and age [*F*(1,60) = 0.083, *P* = 0.775], neither interaction treatment × age [*F*(1,60) = 2.889, *P* = 0.094].

#### Long-Evans Rats

Expression of GluN1 subunits in Long-Evans rats showed no significant main effect of treatment [*F*(1,58) = 0, 41, *P* = 0.524], but a significant main effect of age for administration [*F*(1,58) = 10.062, *P* = 0.002] and significant effect of treatment × age interaction [*F*(1,58) = 6.4068, *P* = 0.014]. Tukey *post hoc* test showed a significant decrease of GluN1 between PD 30 and PD 60 groups with MK-801 (*P* = 0.0013) and between control group PD 30 and MK-801 PD60 (*P* = 0.049). Expression of GluN2B showed no significant main effects of age [*F*(1,52) = 2.6417, *P* = 0.11014] and treatment [*F*(1,52) = 0.375, *P* = 0.543], but a significant treatment × age interaction [*F*(1,52) = 6.1454, *P* = 0.01646]. Tukey *post hoc* test showed a decrease of GluN2B between PD 30 and PD 60 after MK-801 administration (*P* = 0.045). Expression of GluN2A showed no significant main effect of treatment [*F*(1,52) = 0.298, *P* = 0.588], age [*F*(1,52) = 0.014, *P* = 0.907], or interaction [*F*(1,52) = 0.80640, *P* = 0.37333]. However, not significant differences between MK-801- and saline-treated PD 30 and PD 60, respectively, Long-Evans rats were found.

## Discussion

### General Remarks

In this study, we evaluated the effects of a 2-week repeated treatment with the NMDA antagonist MK-801 given in the adolescent and early adulthood periods in Wistar and Long-Evans rats. We detected an elevation of anxiety-like behaviors, measured by time spent in the center and closed arms but not open arms of the elevated plus maze, in Long-Evans rats treated with MK-801 from an adolescent age. Moreover, we found a significant impairment of working memory tested in the MWM in Long-Evans rats treated at the early adult age (from PD 60). No differences were found in cognitive learning and flexibility tested in the Carousel. There were also no significant changes in the expression of NMDA receptor subunits because of MK-801 in any strain and age. Acute administration of NMDA receptor antagonists is used by recreational drug users and in animal models and it can mimic some aspects of psychosis. Abuse of NMDA antagonists in adolescence is associated with higher overall effect, risks for developing psychosis and dependence compared to adult use in human subjects. This notion was demonstrated in rats too (Rocha et al., 2017) where adolescent rats displayed more pronounced behavioral activation to single dose of PCP and ketamine. Contrarily, repeated intermittent dosing of these drugs showed more pronounced sensitization effect in adults than in adolescent rats.

Chronic administration of MK-801, PCP and ketamine in rodent models are currently used to achieve long-term changes in behavior and neurotransmission. Abdul-Monim et al. (2006) reported that female rats trained to solve a simple task failed to complete the task again after chronic PCP administration. Further, the administration of atypical antipsychotics (ziprasidone, olanzapine, clozapine) improved performance, but classical antipsychotics did not have the same beneficial effect (Abdul-Monim et al., 2006). In another study, rats performed significantly worse in the MWM 2 weeks after subchronic MK-801 administration, and at the same time did not differ in weight or locomotor activity (Li et al., 2011).

### Behavioral Changes after Repeated MK-801 Administration

No clear effects on anxiety, working memory, or spatial learning were detected in Wistar PD 30 or PD 60 rats given MK-801 for 2 weeks. In contrast, Long-Evans rats were more affected by this treatment. Our results show that Long-Evans rats from the older age group, i.e., PD 60 at the onset of treatment display cognitive impairments, whereas adolescent Long-Evans rats are susceptible to elevations in anxiety-like behaviors, induced by chronic NMDA receptor antagonism.

#### Anxiety-Like Behavior

We did not detect any alterations in anxiety-like behavior in Wistar rats. Long-Evans rats administered MK-801 on PD 30 displayed higher anxiety levels in the EPM by spending more time in the closed arms than the control group. However, there was no significant change in the time spent in open arms. Studies on long-term changes in anxiety-like behavior due to MK-801 and other glutamatergic psychotomimetics are rather scarce. In contrast to the increased anxiety in PD 30 Long-Evans rats found in this study, Kocahan et al. (2013) and Latysheva and Rayevsky (2003) found decreased levels of anxiety-like behavior in the EPM in Wistar rats treated from PD 7 to PD 10 with 0.25 mg/kg and from PD 7 to PD 49 with 0.05 mg/kg. However, Baier et al. (2009) reported increased anxiety-like behavior on PD 90 in the EPM in Wistar rats after 0.25 mg/kg of MK-801 administered from PD 6 to PD 21. These discrepancies are likely caused by a different dose and timing of applications, but our results imply a susceptibility to elevated anxiety in Long-Evans rats treated in adolescence.

#### Spatial Working Memory in the MWM and Cognitive Learning in the Carousel

Interestingly, testing in the MWM revealed an impairment of working memory only in older PD 60 MK-801-treated Long-Evans rats compared to saline-treated subjects. Rats given MK-801 were still able to learn the task but at a slower rate. Active place avoidance testing did not reveal any difference between MK-801-treated and saline-treated rats 30 days after application, with no effects found on cognitive coordination and flexibility. It should be noted that overall performance of both control and MK-801 rats (Vales and Stuchlik, 2005; Stuchlik et al., 2008), which implies that chronic stress caused by injections in all groups may have negatively affected their performance.

Previously published studies have reported contrasting effects of chronic MK-801 treatment in the postnatal and early adolescent periods on cognition in the spatial and non-spatial domains. McLamb et al. (1990) observed no impairment in a water maze after 0.2 mg/kg MK-801 treatment administered from PD 9 to PD 15 in Fisher-344 rats. Kocahan et al. (2013) failed to find any deficit in an inhibitory avoidance task in Wistar rats treated in the early postnatal age and tested at adolescence (application: PD 7 – PD 10; testing at PD 35 – PD 45). Rats treated two times a day for 7 days with 0.5 mg/kg MK-801 in adulthood were not affected by the treatment in a variable-delayed alternation task or in a T-maze 36 h after administration (Seillier and Guiffrida, 2009). However, other studies have shown clear impairments in spatial memory after chronic MK-801 application, further supporting our data. After 0.25 mg/kg MK-801 given from PD 8 to PD 19, Wistar rats displayed a slower learning rate in a water maze task in adulthood (Gorter and de Bruin, 1992). Latysheva and Rayevsky (2003) treated Wistar rats with 0.05 mg/kg MK-801 from PD 7 to PD 49, and found impaired performance in a complex maze 5 days after treatment. Kawabe and Miyamoto (2008) found impairment in a delayed non-matching-to-position task in adulthood after 0.2 mg/kg and 0.4 mg/kg MK-801 applications two times a day from PD 7 to PD 20 in Wistar rats. In mice, 0.1 mg/kg MK-801 treatment administered from PD 3 to PD 17 worsened performance in the MWM (Elhardt et al., 2010). Finally Li et al. (2011) observed a disruption of spatial working memory in the MWM after 2-week administration OF MK-801 to adolescent Sprague-Dawley rats (starting on PD 28), further indirectly supporting our data although with a different age and strain. Again, different doses and time of administration precludes direct comparison or data of ours and other authors, but the results show a spatial working memory deficit due to MK-801 treatment in young adult Long-Evans rats, supporting the face validity of this treatment in this strain.

#### Locomotor Activity

We did not observe hyperlocomotion in the MWM or active place avoidance after repeated MK-801 treatment, though such an effect has been seen after the acute administration of the same dose (Hargreaves and Cain, 1995; Lobellova et al., 2013; Svoboda et al., 2015). Hyperlocomotion is thought to correlate with the positive symptoms of schizophrenia and is one of the well-characterized effects of chronic MK-801 treatment (Lim et al., 2012). Kocahan et al. (2013) observed increased spontaneous locomotor activity in an open-field test in Wistar rats after 0.25 mg/kg MK-801 given from PD 7 to PD 10. Facchinetti et al. (1993) also observed hyperlocomotion in an open-field test in Wistar rats after the administration of increasing 0.5 – 1 mg/kg doses from PD 1 to PD 22, and this increased activity lasted until PD 60. After 0.25 mg/kg MK-801 administered from PD 6 to PD 21, Schiffelholz et al. (2004) detected increased spontaneous activity on PD 30, but from PD 60 rats displayed hypolocomotion lasting until PD 180. In contrast, however, numerous other authors have reported no effects of early-life MK-801 treatment on spontaneous locomotor activity in rats (Stefani and Moghaddam, 2005; Kawabe et al., 2007; Uehara et al., 2009). McLamb et al. (1990) reported no effects of 0.2 mg/kg MK-801 administered from PD 9 to PD 15 in Fischer-344 rats on locomotor activity in the MWM. The absence of locomotor changes in our model suggests that it does not constitute a model of positive symptoms of schizophrenia.

### Effects of Repeated Administration of MK-801 on NMDA Receptor Subunits

We did not detect any significant changes in the expression of GluN1, GluN2A, or GluN2B subunits in MK-801-treated Wistar or Long-Evans rats compared with control groups. We only observed differences between different times of administration of MK-801. In accordance with our data, Wang et al. (1999) failed to detect any change in GluN1 subunit expression in the hippocampus of rats chronically administered with PCP, and only found an increase in the GluN1 subunit in the forebrain. Chronic PCP given to adult mice resulted in an increased number of binding sites for MK-801 shortly after treatment (Newell et al., 2007). However, 14 days after treatment the number of MK-801 binding sites decreased significantly, especially in the hippocampus. These results are in partial accordance with our present data. However, Oh et al. (2001) reported increased numbers of the GluN1 subunit in CA1 of the hippocampus after intracerebroventricular infusion with MK-801 in Sprague-Dawley rats. Anastasio and Johnson (2008) reported an increased number of GluN1 and GluN2B subunits induced by translocation from the endoplasmic reticulum to the membrane after acute PCP administration. In contrast, repeated administration of PCP led to the increased *de novo* synthesis of NMDA subunits. It has to be pointed out that the amounts of protein for NMDA receptor subunits might not be equal to the number of active receptors, which may contributed to the negative findings reported in this study. The changes between Long-Evans rats treated from PD 30 vs. PD 60 may have been a result of different brain responses in relation to the age of the rats during the administration of MK-801. Matta et al. (2013) described the disruption of developmental changes in NMDA receptors induced by giving MK-801 during ontogenesis. Age-dependent differences may also occur due to different NMDA currents in immature neurons compared to fully mature neurons and due to the different ontogenetic development of individual structures (Wang and Gao, 2009; Rotaru et al., 2011; Nakazawa et al., 2017). GluN2B and GluN2A subunits of the NMDA receptor show varied expression during ontogenetic development. The GluN2B subunit is most highly expressed in the first PDs and its concentration gradually decreases, while GluN2A concentrations are low after birth and gradually rise until PD 21, when they reach levels comparable with those in adulthood (Wenzel et al., 1997). Our finding that in MK-801-treated animals the levels of GluN1 and GluN2B are changed due to age of treatment most likely represent the developmental changes in sensitivity of NMDA receptor system to this drug. The absence of MK-801-induced effects also decreases the construct axis of validity of this model.

### Limitations of the Study

This study has a few limitations. Notably, both strains were testing in different batches as mentioned in the section “Materials and Methods,” preventing their direct comparison. In addition, both age cohorts underwent their behavioral testing at different periods of their development (PD 49 – PD 69 vs. PD 78 – PD 97), but intended to keep the interval between administration of the drugs and behavioral testing constant to allow for comparison. Repeated drug administration in the adolescent period raises concerns regarding the correct physical development of the animal. However, in this study, rats treated with MK-801 did not differ in total health status or reactions to environmental stimuli during behavioral testing (visual observations; data not shown) except the three rats that exhibited aggressiveness that were excluded from the study.

## Conclusion

Chronic treatment with the NMDA antagonist MK-801 impaired working memory only in Long-Evans rats treated in early adulthood with application starting from PD 60. Suggestively elevated anxiety was found in Long-Evans rats treated at adolescent age. This data support the face validity of this treatment as potential animal model of cognitive deficits due to chronic experimental psychosis. However, no significant effects were observed in expression of NMDA subunits by Long-Evans or Wistar strains at any age. These finding did not support the construct validity of this model. In conclusion, our results suggest that despite a working memory deficit and elevated anxiety in one of the strains, this dose, timing and period of treatment with MK-801 does not constitute a plausible model of schizophrenia-like phenotypes.

## Author Contributions

All authors contributed to writing of the manuscript. HS, MS, and VL conducted the behavioral study. LU and GT conducted the Western blot. AS conceived the study and provided the scientific leadership.

## Conflict of Interest Statement

The authors declare that the research was conducted in the absence of any commercial or financial relationships that could be construed as a potential conflict of interest.

## Abbreviations

ANOVA: analysis of variance
EPM: elevated-plus maze
MK-801: dizocilpine maleate
MWM: Morris water maze
NMDA (receptors): *N*-methyl-D-aspartate (subtype of glutamate receptors)
PCP: phencyclidine
PD: postnatal day
SEM: standard error of the mean
TBS: Tris-buffered saline
