# Corrigendum: Chronic MK-801 Application in Adolescence and Early Adulthood: A Spatial Working Memory Deficit in Adult Long-Evans Rats But No Changes in the Hippocampal NMDA Receptor Subunits

**DOI:** 10.3389/fphar.2018.00693

**Published:** 2018-06-20

**Authors:** Libor Uttl, Tomas Petrasek, Hilal Sengul, Marketa Svojanovska, Veronika Lobellova, Karel Vales, Dominika Radostova, Grygoriy Tsenov, Hana Kubova, Anna Mikulecka, Jan Svoboda, Ales Stuchlik

**Affiliations:** ^1^Department of Developmental Epileptology, Institute of Physiology, Czech Academy of Sciences, Prague, Czechia; ^2^Department of Experimental Neurobiology, National Institute of Mental Health, Klecany, Czechia; ^3^Department of Neurophysiology of Memory, Institute of Physiology, Czech Academy of Sciences, Prague, Czechia; ^4^Radboud Institute for Molecular Life Sciences, Radboud University, Nijmegen, Netherlands; ^5^Second Faculty of Medicine, Charles University, Prague, Czechia

**Keywords:** schizophrenia, animal model, dizocilpine, rats, chronic treatment, western blot, behavior

There is an error in the Funding statement. The correct number for **OPPK CZ.2.16/3.1.00/** is **OPPK Microscopic System CZ.2.16/3.1.00/28034**. The authors apologize for this error and state that this does not change the scientific conclusions of the article in any way.

The original article has been updated.

## Conflict of interest statement

The authors declare that the research was conducted in the absence of any commercial or financial relationships that could be construed as a potential conflict of interest.

