# Supplementary material for: Chronic MK-801 Application in Adolescence and Early Adulthood: A Spatial Working Memory Deficit in Adult Long-Evans Rats But No Changes in the Hippocampal NMDA Receptor Subunits
Source: Front Pharmacol. 2018 Feb 12;9:42. doi: 10.3389/fphar.2018.00042 (PMC5816576; doi:10.3389/fphar.2018.00042)

Supplementary Material:

A) 4-15 % Criterion Stain-Free gel (5678084)

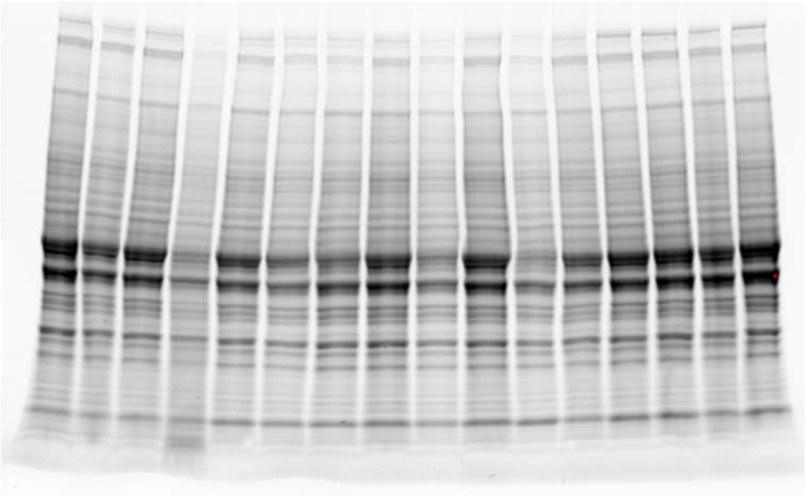

B) Stain-Free Membrane after transfer *(170-4271, Bio-Rad*) – for normalization

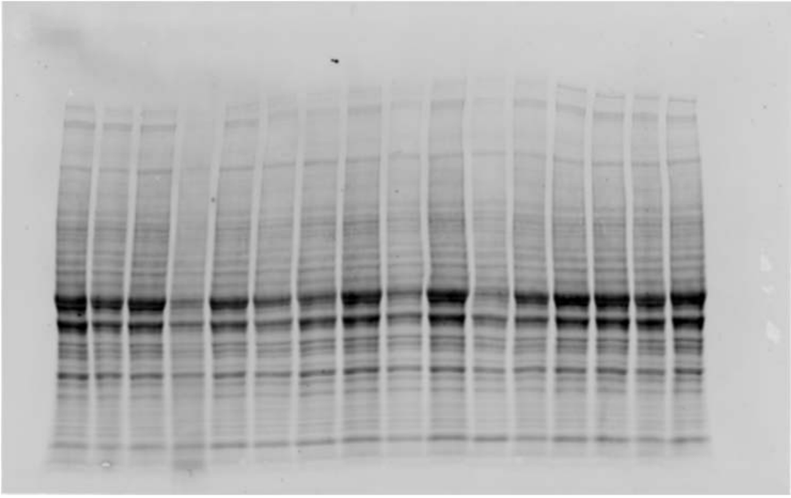

C) Molecular weight – C.1: kDa standard that was used (#1610374, Bio-Rad), C.2: colorimetric image of membrane, C.3: stain-free membrane (the same as C.2.)

C.1 C.2 C.3

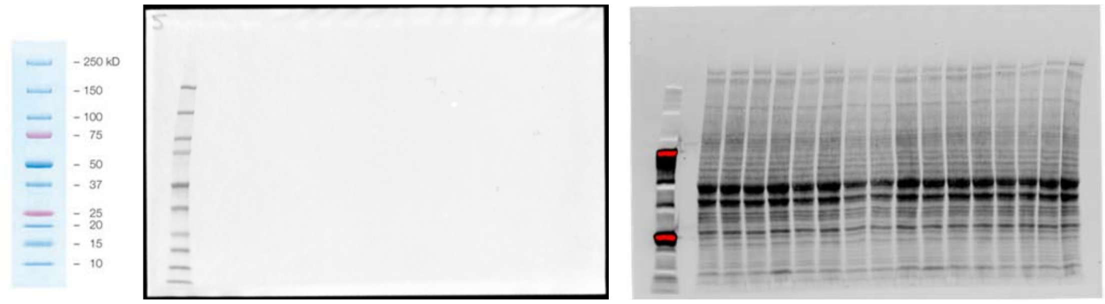

D) Sequences of samples on membranes

rat strains	designation of membranes	Lane 1	Lane 2	Lane 3	Lane 4	Lane 5	Lane 6	Lane 7	Lane 8	Lane 9	Lane 10	Lane 11	Lane 12	Lane 13	Lane 14	Lane 15	Lane 16	Lane 17	Lane 18
gels MS LongEvans rats	(NR2A) MS 15, MS 16, MS 17, MS 18<br>(NR1) MS 1, MS 4, MS 6, MS 11<br>(NR2B) MS 3, MS 8, MS 10, MS 12	Free lane or kDa standard	C -30	C -30	C -30	C -30	M-30	M-30	M-30	M-30	C-60	C-60	C-60	C-60	M-60	M-60	M-60	M-60	Free Lane
gel with Wistar rats samples	(NR2B) membrane L, membrane I	Free lane or kDa standard	C -30	C -30	C -30	C -30	M-30	M-30	M-30	M-30	C -30	C -30	C -30	C -30	M-30	M-30	M-30	M-30	Free Lane
	(NR2B) membrane C, membrane F		C-60	C-60	C-60	C-60	M-60	M-60	M-60	M-60	C-60	C-60	C-60	C-60	M-60	M-60	M-60	M-60	
	(NR1) membrane 1, membrane 9		C -30	C -30	C -30	C -30	M-30	M-30	M-30	M-30	C -30	C -30	C -30	C -30	M-30	M-30	M-30	M-30	
	(NR1) membrane A, membrane D, membrane 5 membrane 10,		C-60	C-60	C-60	C-60	M-60	M-60	M-60	M-60	C-60	C-60	C-60	C-60	M-60	M-60	M-60	M-60	
	(NR2A) membrane 6, membrane 7		C -30	C -30	C -30	C -30	M-30	M-30	M-30	M-30	C -30	C -30	C -30	C -30	M-30	M-30	M-30	M-30	
	(NR2A) membrane 2, membrane 8		C-60	C-60	C-60	C-60	M-60	M-60	M-60	M-60	C-60	C-60	C-60	C-60	M-60	M-60	M-60	M-60	

abbreviations	
	C-30, control (start of administration PD30)
	C-60, control (start of administration PD60)
	M-30, MK-801 (start of administration PD30)
	M-60, MK-801 (start of administration PD30)

E) Original membranes

Long-Evans - GluN1

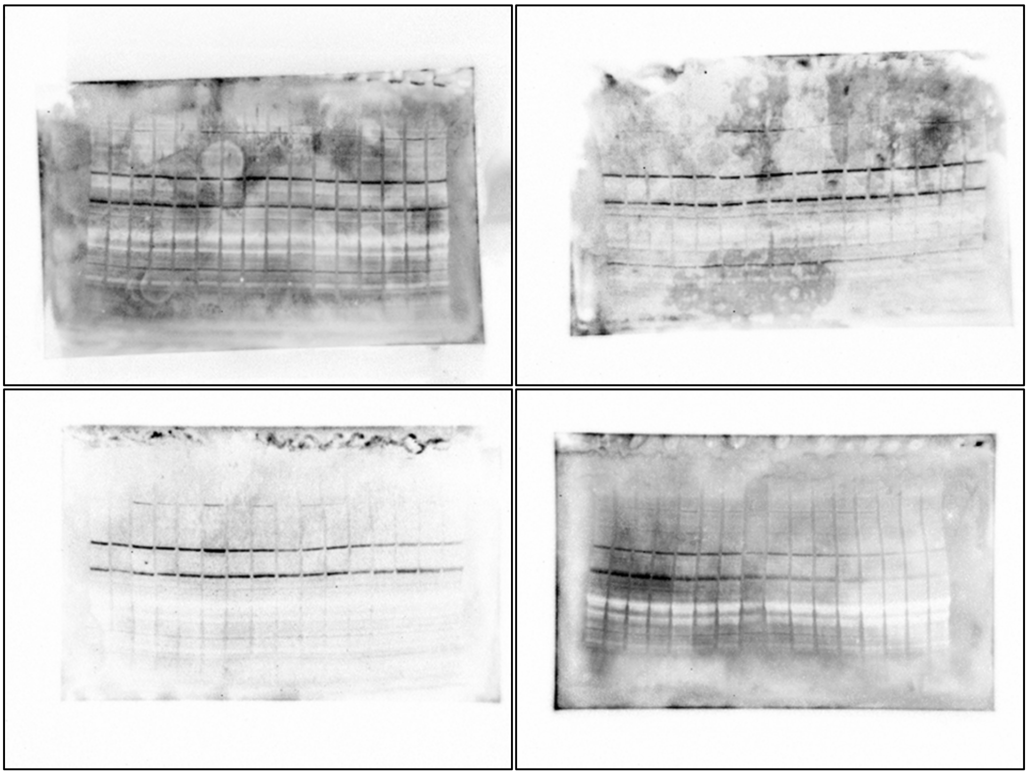

Long-Evans - GluN2A

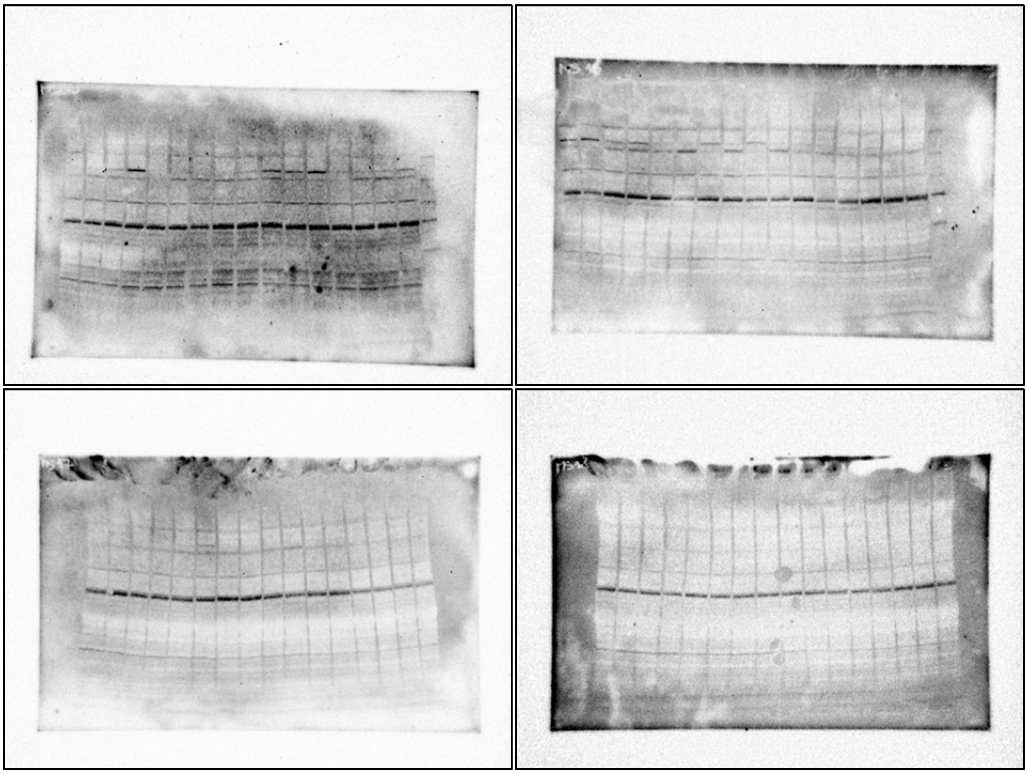

Long-Evans - GluN2B

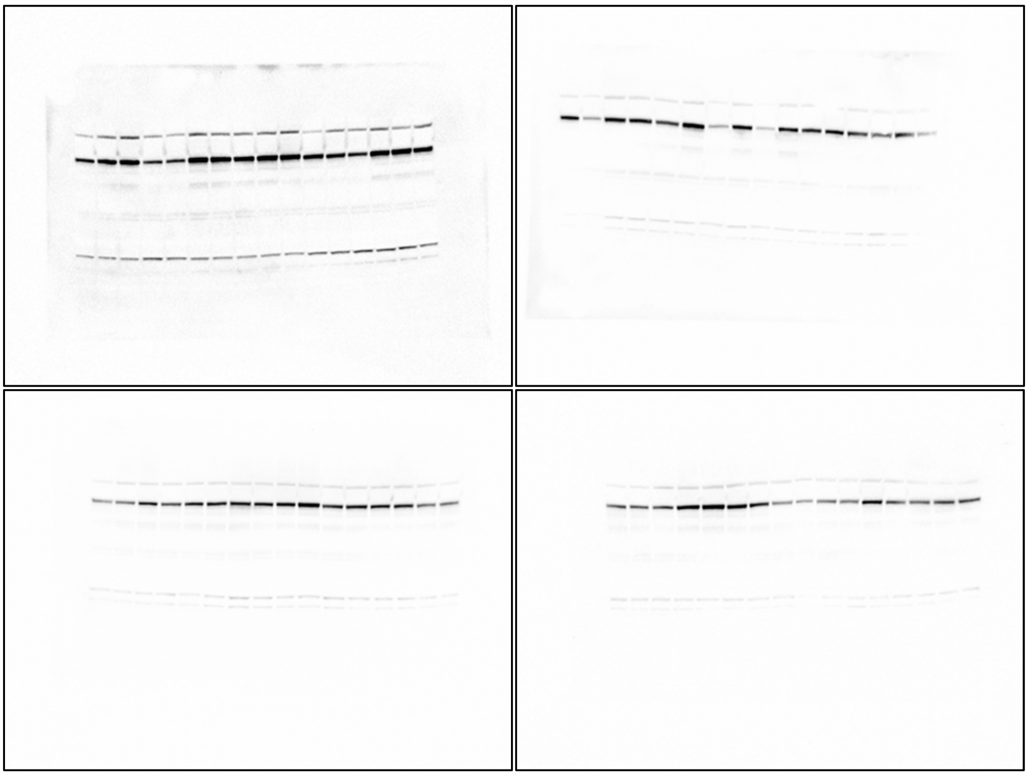

Wistar - GluN1

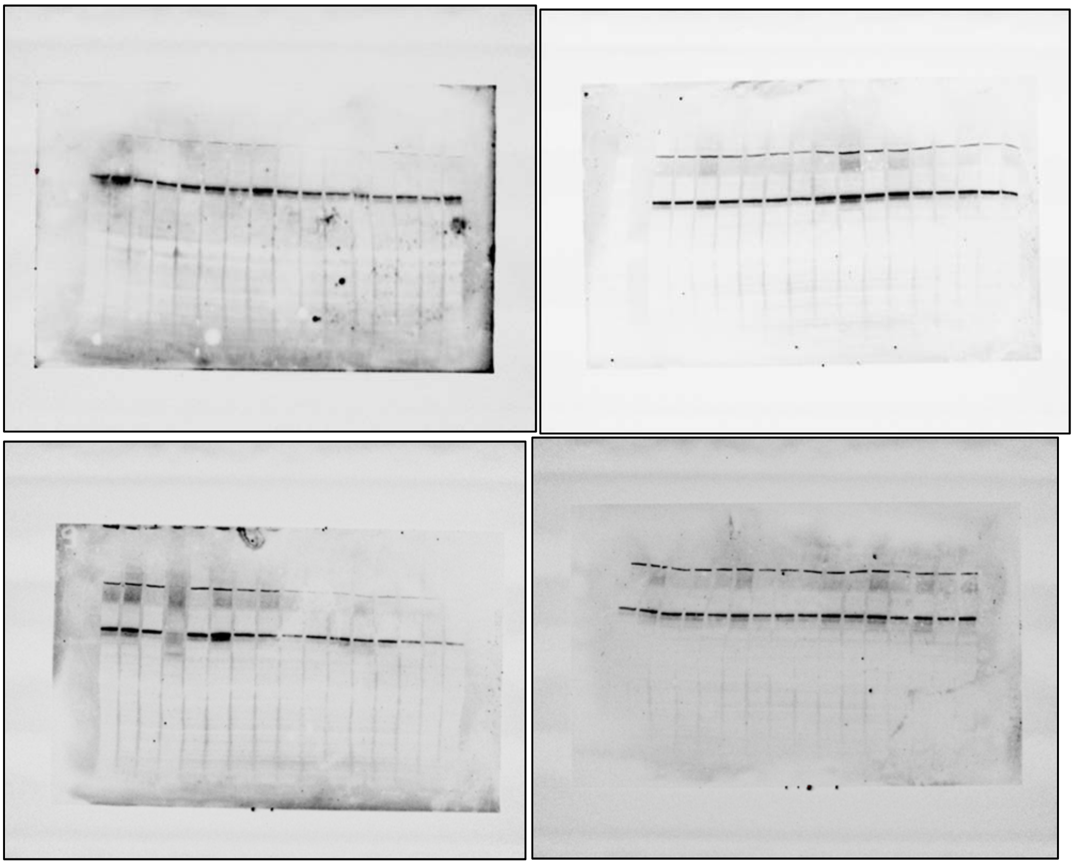

Wistar – GluN2A

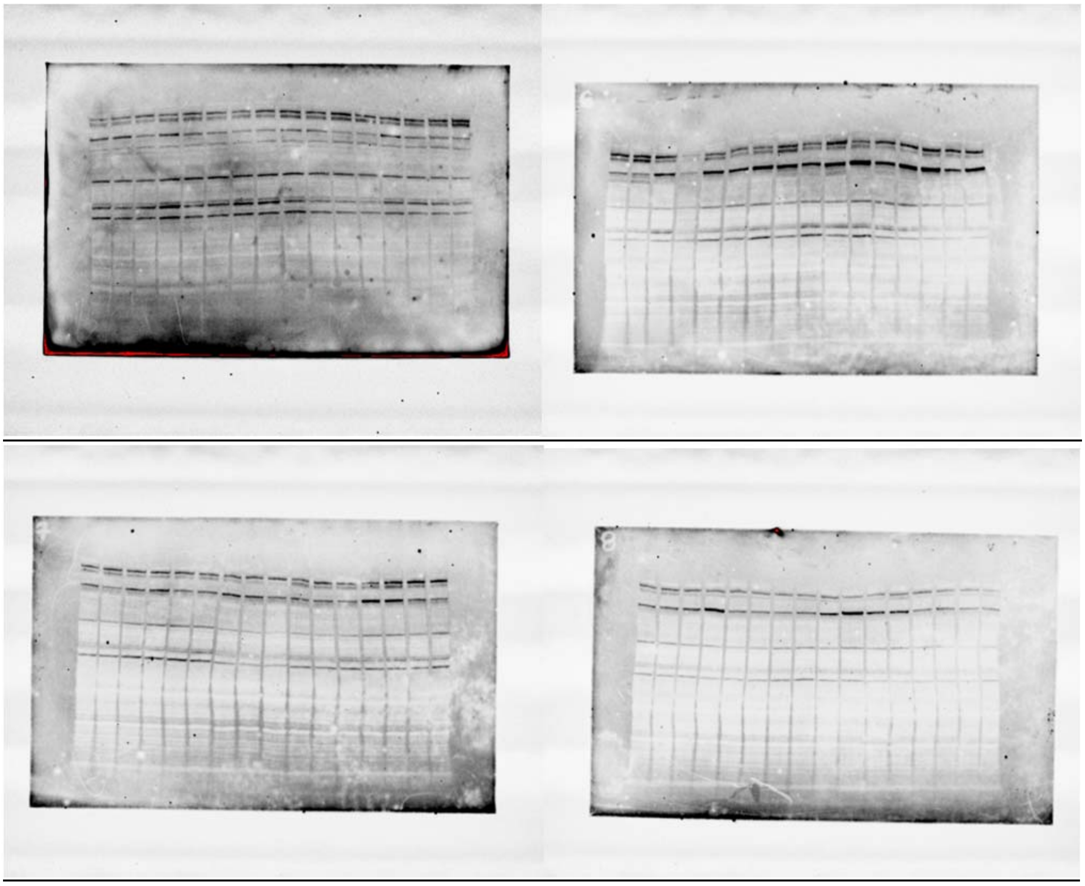

Wistar - GluN2B

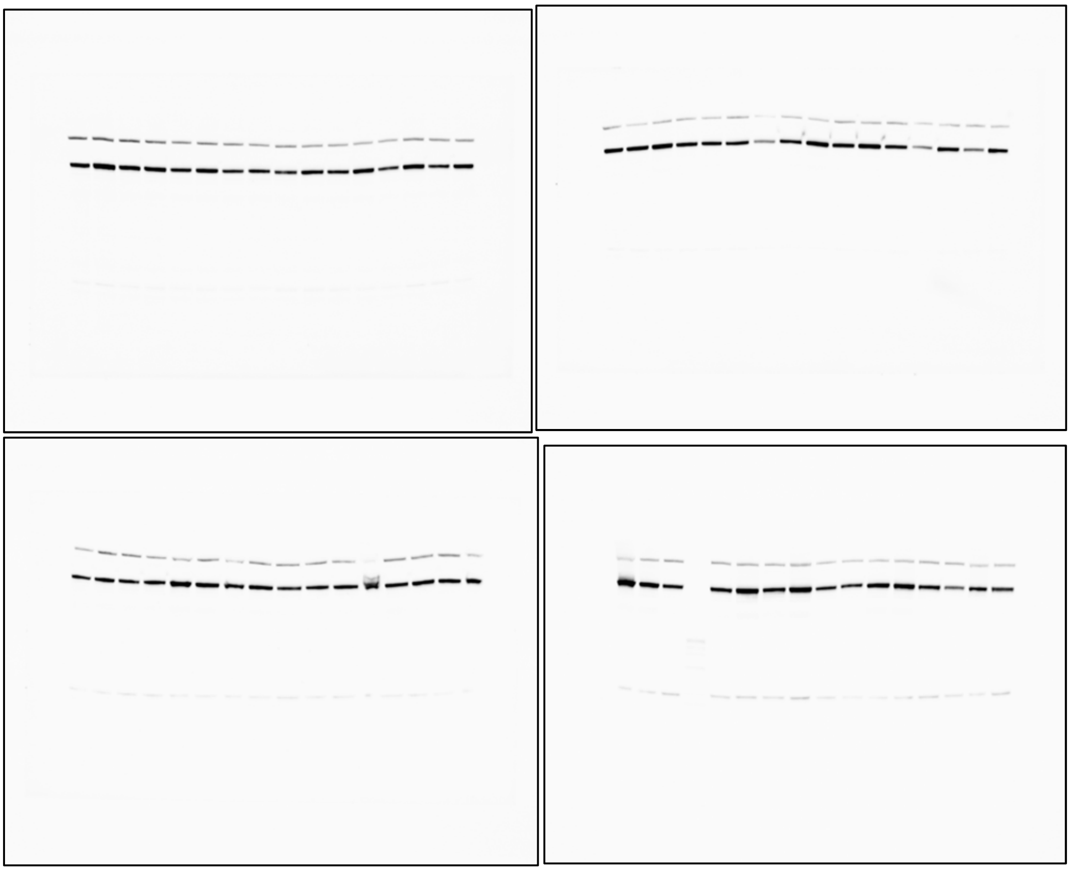

Supplement: Supplementary file 1 [file Image_1.PDF]
